# A new method of software vulnerability detection based on a quantum neural network

**DOI:** 10.1038/s41598-022-11227-3

**Published:** 2022-05-16

**Authors:** Xin Zhou, Jianmin Pang, Feng Yue, Fudong Liu, Jiayu Guo, Wenfu Liu, Zhihui Song, Guoqiang Shu, Bing Xia, Zheng Shan

**Affiliations:** 1 State Key Laboratory of Mathematical Engineering and Advanced Computing, Zhengzhou, 450000 Henan China; 2 Songshan Laboratory, Zhengzhou, 450000 Henan China; 3 State Key Laboratory of Complex Electromagnetic Environment Effects on Electronics and Information System, Luoyang, 471000 Henan China

**Keywords:** Computer science, Information technology, Quantum information

## Abstract

In the field of network security, although there has been related work on software vulnerability detection based on classic machine learning, detection ability is directly proportional to the scale of training data. A quantum neural network has been proven to solve the memory bottleneck problem of classical machine learning, so it has far-reaching prospects in the field of vulnerability detection. To fill the gap in this field, we propose a quantum neural network structure named QDENN for software vulnerability detection. This work is the first attempt to implement word embedding of vulnerability codes based on a quantum neural network, which proves the feasibility of a quantum neural network in the field of vulnerability detection. Experiments demonstrate that our proposed QDENN can effectively solve the inconsistent input length problem of quantum neural networks and the problem of batch processing with long sentences. Furthermore, it can give full play to the advantages of quantum computing and realize a vulnerability detection model at the cost of a small amount of measurement. Compared to other quantum neural networks, our proposed QDENN can achieve higher vulnerability detection accuracy. On the sub dataset with a small-scale interval, the model accuracy rate reaches 99%. On each subinterval data, the best average vulnerability detection accuracy of the model reaches 86.3%.

## Introduction

With the rapid development and popularization of 5G networks, network security is still a key issue to be solved in the industry, including malware analysis and vulnerability detection^1–3^. As the core issue, there have been researches such as static and dynamic analysis on vulnerability detection in the past few years. Among them, static analysis includes vulnerability detection based on rule matching, code comparison, and symbolic execution^4–6^. These methods do not require running programs and can efficiently and quickly complete amounts of program code. However, with increasing software complexity, it is not suitable for large-scale unknown vulnerabilities^7^. Dynamic analysis includes fuzzing^8,9^ and taint analysis^10,11^, but these methods have the problem of low path coverage, path explosion and consumes extensive computing resources.

It is worth noting that with the rise of machine learning technology, vulnerability detection based on machine learning has become a hot issue. At present, this field includes attribute-based software code measurement^12^, code similarity detection^13–15^, etc. To detect the unknown vulnerability in an actual application environment, the combination of word embedding and vulnerability detection came into being. Instruction2vec^16^ proposed for assembly instruction word embedding combined with the Text-CNN model to achieve vulnerability analysis. An instruction embedding method based on variational autoencoders has been proposed and named MDSeqVAE^17^. It is worth noting that in the motivating research^18^, a novel intermediate code presentation “code gadget” was proposed. A code gadget consists of multiple lines of code that are semantically related and can be extracted via a program slicing technique. This work uses code gadgets as code representations and implements vulnerability detection based on BLSTM. Furthermore, they^19,20^ have confirmed that it is appropriate to perform vulnerability detection on code gadgets because each code gadget is associated with key points (for example, API calls) that indicate vulnerabilities. Although previous works over the past few years have investigated the effectiveness of code gadgets, there are indeed differences in quantum computing, so fully transfer is not suitable. In the motivating research^18^, they implement vulnerability detection based on the classic BLSTM. However, the classic LSTM corresponding QLSTM^21,22^ model has obvious shortcomings. In the QLSTM, a large number of measurement operations are required in the implementation process, which greatly increases the time cost. We analyze it in detail in “Analysis of variable length data processing” section. Although classic machine learning has been proven to be applicable to vulnerability detection, there are certain limitations in this research. Vulnerability detection capability based on neural networks is directly proportional to the scale of the training data, and the expansion of training data will also lead to an increase in the cost of neural network training. Furthermore, the expansion of a classical neural network structure will cause a storage performance bottleneck in a classical computer. Therefore, there are still challenges and difficulties in solving vulnerabilities based on classic machine learning.

Quantum computing is based on the postulates and characteristics of quantum mechanics (i.e., quantum bits (qubits), interference, superposition, and entanglement) for information processing. A qubit can have a one state, zero state, or a combination of two states at the same time, which is known as linear superposition, unlike a classical bit, which can represent one value, either 0 or 1, to store information^23^. Quantum computing solves the storage performance bottleneck problem of classic computers. Quantum machine learning (QML) techniques are more effective in many real-world applications than traditional machine learning in both speed and accuracy^24^.

Currently, quantum machine learning has made progress in related fields, such as natural language processing^21,25–29^, recommendation systems^30,31^, speech recognition, image classification, and the medical domain. QML can improve the running time and efficiency of programs. It can obtain higher performance than classic deep learning and traditional algorithms through low-cost big data training data^32,33^, which provides solutions to the above problems.

To the best of our knowledge, there is no research on the use of quantum neural networks for vulnerability detection. Quantum neural networks have been proven to solve the memory bottleneck problem of classical machine learning, so they have far-reaching prospects in the field of vulnerability detection. This work is the first to implement vulnerability data word embedding based on a quantum neural network, which proves the feasibility of a quantum neural network in the field of vulnerability detection.

The contributions of this work can be summarized as follows:

1. There have not yet been combined and applied in vulnerability detection with quantum neural networks. To fill this gap, we propose a new quantum neural network structure, the quantum deep embedding neural network (QDENN), for vulnerability detection.
2. Compared with classical neural network, quantum neural networks can process classical information at a small memory consumption, taking advantage of the characteristics of quantum mechanics. Therefore, in addition to verifying the feasibility of vulnerability detection based on quantum neural networks, this work shows the far-reaching prospects of network security applications based on quantum neural networks.
3. In the field of large-scale vulnerability detection, vulnerability programs have longer lengths, and the lexical structure is more complex. Experimental results demonstrated that, in the field of vulnerability detection, our proposed QDENN can effectively solve the problem of inconsistent input lengths of quantum neural networks. Furthermore, it can give full play to the advantages of quantum computing and realize a vulnerability detection model at the cost of a small amount of measurement.
4. Although there have been related works on NLP based on quantum neural networks, they generate quantum circuits for each sentence based on tensor networks. However, this is not suitable for sophisticated vulnerability detection. Our proposed QDENN can also solve the problem of batch processing with long sentences.
5. Compared to other quantum neural networks, the proposed QDENN can achieve higher vulnerability detection accuracy. In the sub dataset of a small-scale interval, the model accuracy rate reaches 99%. In each subinterval data, the best average vulnerability detection accuracy of the model reaches 86.3%.

## Results

### The advantage of a quantum neural network

Large-scale neural network training is a computationally intensive task, and memory is one of its biggest challenges. Existing solution require amounts of custom silicon, additional memory and computational resources^34^. Therefore, existing structure named “von Neumann” has become a significant bottleneck of classical neural networks.

The emergence of quantum computing provides a solution to the structural bottleneck problem. Quantum computers are based on quantum entanglement and quantum superposition states. Compared with the binary-represented crystals in classical computers, qubits have stronger data representation capabilities, which is why they can theoretically achieve higher computing capabilities. Since qubits operate in a completely different way from classical computers, there will also be no so-called memory bottlenecks.

Related research^35^ has shown that compared with classical computing, quantum neural networks have rich prospects. Meanwhile, the research^36^ quantitatively studied the computational advantages of quantum neural networks through theoretical derivation. Cutting-edge research^37,38^ explained the quantum advantage from the capacity of models.

Compared with classical neural networks, quantum neural networks can achieve significantly higher effective dimensions. Furthermore, quantum neural networks with higher effective dimensions are trained to lower loss values in fewer iterations, meaning that they can also fit data well.

Therefore, quantum neural networks have been proven to solve the memory bottleneck problem of classical machine learning, so it has far-reaching prospects in the field of vulnerability detection.

### Model overview

The motivation of our work is to demonstrate the feasibility of quantum neural networks in vulnerability detection, which could solve the memory bottleneck problem of classic computers. Our goal is to provide a program in which a quantum neural network can provide vulnerability detection results and the location of a vulnerability. According to the relevant research^39^, it proposes a definition method of vulnerability patterns and patch patterns for binary files. Based on the results of the taint analysis, they define vulnerability patterns in the form of triples. Specifically, it defines the vulnerability pattern as <source, sink, sanity check>. Among them, source refers to the pollution source of user or external input, sink refers to the relevant operation involving the pollution source, and sanity check refers to the integrity check of the operation performed on the input. Therefore, we can roughly define the concept of vulnerability as uncontrollable input sources and dangerous uses. Among them, dangerous uses can be embodied as arrays, API calls, etc. According to analysis and observation, insecure API calls often cause vulnerabilities. As a result, we take an API call as the research object of this work and slice the relevant code to form code gadgets by slicing the key points of vulnerability triggering. Through the word segmentation encoding of the code gadgets, we realize the vulnerability detection based on a quantum neural network, using the expected values of the Z_0_ and Z_1_ hamiltonian.

**Figure 1:**
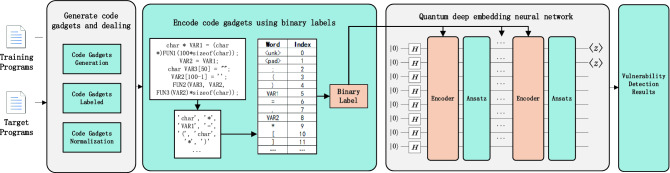
The framework for using a quantum neural network to detect vulnerabilities. The centre represents the overall structure of the three parts. **(a)** Generating and dealing with code gadgets. Generate code gadgets by slicing the relevant code according to the API functions and dealing with standardized processes. **(b)** Encoding code gadgets using binary labels. The code gadgets are encoded using binary labels, and the obtained word vector is used as the input to the quantum neural network. **(c)** Quantum deep embedding neural network. The value of each word vector is encoded in the quantum neural network. Based on quantum circuit simulation, the probability result of vulnerability detection is finally output through measurement.

Figure 1 shows the model architecture of this paper. It can be divided into three parts: the generation of code gadgets, binary label representation, and a quantum deep embedding neural network. Next, we elaborate on the proposed model in detail.

We regard API calls as the research object of this work, and code gadgets can be generated based on data flow or control flow. Although each vulnerability may be associated with multiple vulnerability functions, in this paper, we only consider the case where a vulnerability is associated with one function.

**Figure 2:**
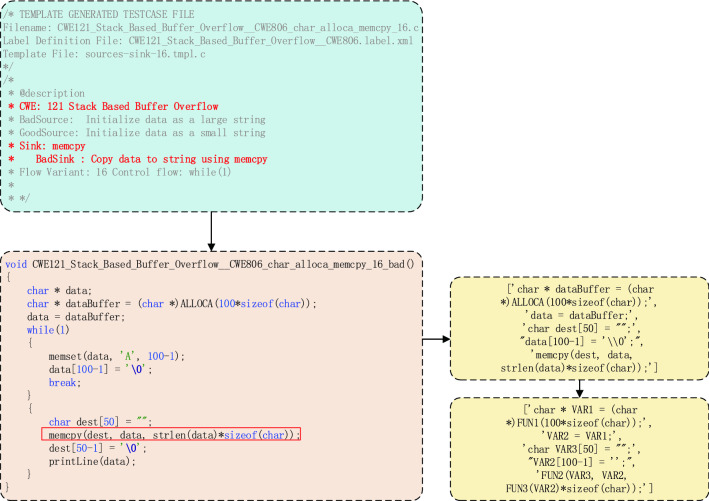
The example of extracting code gadgets. To illustrate the extraction of code gadgets, we choose CWE121_Stack_Based_Buffer_Overflow__CWE806_char_alloca_memcpy_16.c as an example. In the flowchart, we generate code gadgets by slicing the relevant code according to the API functions and dealing with standardized processes.

As shown in the Fig. 2, we choose a vulnerability program as the example to illustrate the extraction of code gadgets. First, we locate the library function API according to the vulnerability indication information (such as “BadSink: Copy data to string using memcpy” in the Fig. 2) and then slice the relevant code according to the API. This can be divided into forward API function calls and backward API function calls. A forward API call refers to the API parameters that receive data directly from the socket, and a backward API call refers to the API parameters that do not directly receive data from the socket, such as the stack length setting. For forward API calls, the uncontrollable input source of API parameters is more important. For backward API calls, parameter setting transfer is more important. This corresponds to the rough definition of vulnerabilities, uncontrollable input sources and dangerous use we previously put forward. Based on forward and backward slicing, we can obtain code gadgets.

At the same time, according to the vulnerability indication information, each code gadget is labelled. If the code gadgets have vulnerabilities, they are marked as 1; otherwise, they are marked as 0.

Next, the API is pre-processed and standardized. First, we delete non-ascii characters and comments in the source code. Then, with code gadgets as the unit, the variable names are standardized to “VAR1”, “VAR2”, etc. according to their order of appearance. At the same time, the function names are standardized to “FUN1”, “FUN2”, etc. according to their order of appearance. In this way, the generalization ability of the model can be improved, and the quantum neural network can learn the causes of vulnerabilities instead of only learning the causes of specific API function calls. Finally, we obtain code gadgets for vulnerability detection.

Next, we construct a dictionary based on the obtained code gadgets, encode the vulnerable source code segmentation according to the binary labeled method, and input it into the next quantum neural network model in the form of quantum state angel encoding.

### Quantum deep neural network

To explain our quantum neural network model more clearly, we define the vulnerability detection problem.

The task of vulnerability detection is to perform supervised learning on the set $L = \left\{ {1,2,...,l} \right\}$ of $l$ labels. Among them, we give a training set $T$ and a validation set $V$, and assume that there is a label mapping relationship $m_{{\left| {T \cup V} \right.}}$($T \cup V \to L$). For the quantum neural network model we propose, both $T$ and $V$, and the mapping relationship of $m_{\left| T \right.}$($T \to L$).

The goal of the quantum neural network model is to train the speculation ability ($\tilde{m}:V \to L$) on the verification set $V$ based on $m_{\left| T \right.}$, that is, to improve accuracy probability $m_{\left| V \right.} \left( {\vec{v}} \right) = \tilde{m}\left( {\vec{v}} \right)$ as much as possible, among which $\vec{v} \in V$.

In the field of quantum computing, a common method is to construct a computable function $\tilde{m}:\left( {\vec{\theta },V} \right) \to L$, in which $\vec{\theta }$ is a set of weight parameters that can be trained. What is different from classical neural networks is that these parameters have physical meanings such as angle information. Consistent with a classic neural network, these parameters can be optimized and trained by constructing a classic cost function.

In our work, we propose a quantum neural network model structure QDENN for vulnerability detection, which is a VQC-based quantum neural network^40^. A VQC model constructs a separating hyperplane in the state space of $n$ qubits. The QDENN is built up to use entanglement from quantum computing to model complex and highly correlated distributions, which is expected to achieve higher performance over a classical neural network.The algorithm consists of two main parts: the training phase and the detection phase.
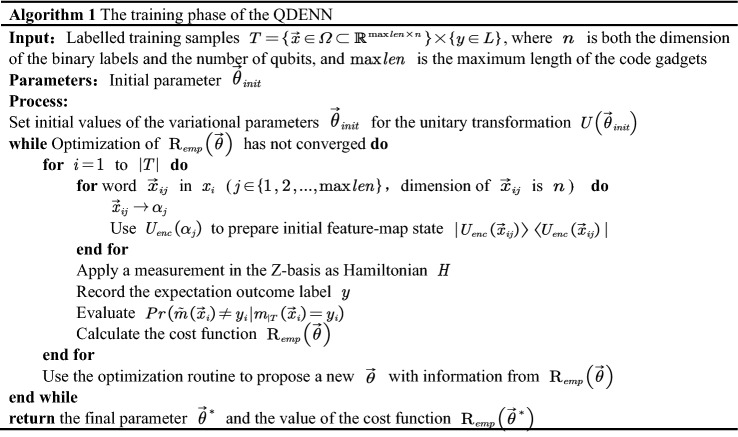

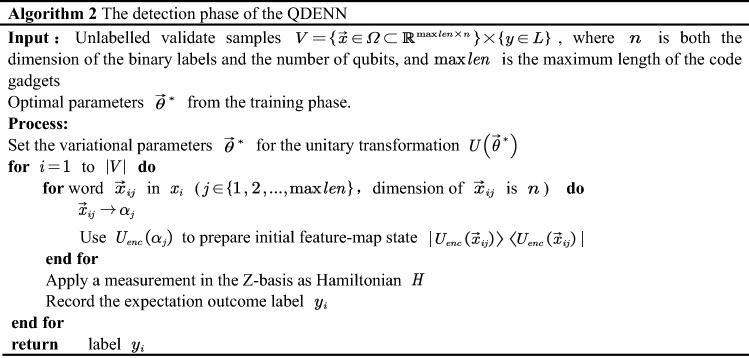


**Figure 3:**
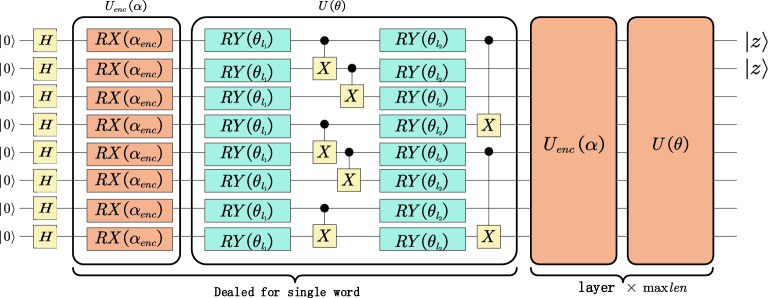
Overview of the quantum deep embedding neural network used in this study. Gate Rx($\alpha$) is the rotation gate generated by Pauli X. According to the algorithm illustrated in this study, the rotation angle is determined using the given classical data x. U(θ) represents a general gate with Ry (the rotation gate generated by Pauli Y) and a controlled X-gate (CNOT-gate). Because the layer is repeated maxlen times, we measure the expectation of Pauli Z based on the first two qubits as the result of the quantum deep embedding neural network.

Figure 3 shows the general framework of the QDENN, which consists of three components: (1) a series of quantum gates with non-adaptable parameters $\alpha$ for state preparation; (2) a series of quantum gates with adaptable parameters $\theta$ to mimic biological neurons; and (3) a set of measurement operations to extract output.

When constructing a quantum neural network structure, quantizing the characteristics of the vulnerable source code is very important. In this section, we first introduce how to encode the word vector obtained in the previous section into quantum state data. Feature mapping is first run through a unitary operator applied to a set of quantum nodes as a method of encoding the classical information in the new qubit space. A unitary matrix, needed to encode the information, must be classically derived before applying it to a quantum circuit. Its parameters are determined by the values of the preceding classical nodes at the point of insertion.

By converting the decimal label to binary representation, we can use $\log_{2} N$ qubits representing the dictionary consists of N words. The quantum encoding method adopt angle encoding, which can be realized based on different gate operations. Therefore, the binary label and angel encoding method occupies O(log(N)) qubit. In this paper, we first apply a Hadamard gate to place the qubits in a superposition state and then apply $R_{x}$ gates to the qubits to rotate the angle to the same eigenvalues previously entered. In this encoding method, there is no entanglement, and classical nodes are merely replaced with a parameter quantum node^41^. In our work, we use $n_{q}$ to represent the number of qubits and $U_{enc} \left( \alpha \right)$ to represent the unitary transformation of the encoding circuit. Meanwhile, there are a series of quantum gates with adaptable parameters $\theta$ to mimic biological neurons after the encoding circuit. We use $U\left( \theta \right)$ to represent the unitary transformation of an ansatz circuit.

The unitary transformation $U_{sw}$ that processes a single word in code gadgets is composed of $U_{enc} \left( \alpha \right)$ and $U\left( \theta \right)$. The relevant calculation formula is as follows:

$$U_{sw} = U\left( \theta \right)U_{enc} \left( \alpha \right),$$

$$U_{enc} \left( \alpha \right) = \otimes_{i = 0}^{{n_{q} }} \left( {R_{x} \left( {\alpha_{i} } \right)} \right),$$

$$U\left( \theta \right) = \otimes_{{i_{1} = 0}}^{{n_{q} }} \left( {R_{y} \left( {\theta_{{i_{1} }} } \right)} \right)\prod\limits_{{i_{2} = 0}}^{{\left\lfloor {n_{q} /3} \right\rfloor }} {CX_{{3i_{2} ,3i_{2} + 1}} } \prod\limits_{{i_{3} = 1}}^{{\left\lfloor {n_{q} /3} \right\rfloor }} {CX_{{3i_{3} - 2,3i_{3} - 1}} } \otimes_{{i_{4} = 0}}^{{n_{q} }} \left( {R_{y} \left( {\theta_{{i_{4} }} } \right)} \right)\prod\limits_{{i_{5} = 0}}^{{\left\lfloor {n_{q} /4} \right\rfloor }} {CX_{{4i_{5} ,4i_{5} + 3}} } .$$

The circuit to process a single word in code gadgets returns the state

$$|\varphi_{i} \rangle = \left\{ {\begin{array}{*{20}l} {U_{sw} H|\varphi_{0} \rangle \, \, ,\,i = 1} \hfill \\ {U_{sw} |\varphi_{i - 1} \rangle \,,\,i > 1} \hfill \\ \end{array} } \right.,$$

$$|\varphi_{i} \rangle = \otimes_{{i_{1} = 0}}^{{n_{q} }} \left( {R_{y} \left( {\theta_{{i_{1} }} } \right)} \right)\prod\limits_{{i_{2} = 0}}^{{\left\lfloor {n_{q} /3} \right\rfloor }} {CX_{{3i_{2} ,3i_{2} + 1}} } \prod\limits_{{i_{3} = 1}}^{{\left\lfloor {n_{q} /3} \right\rfloor }} {CX_{{3i_{3} - 2,3i_{3} - 1}} } \otimes_{{i_{4} = 0}}^{{n_{q} }} \left( {R_{y} \left( {\theta_{{i_{4} }} } \right)} \right)\prod\limits_{{i_{5} = 0}}^{{\left\lfloor {n_{q} /4} \right\rfloor }} {CX_{{4i_{5} ,4i_{5} + 3}} } \otimes_{{i_{6} = 0}}^{{n_{q} }} \left( {R_{x} \left( {\alpha_{{i_{6} }} } \right)} \right) \, |\varphi_{i - 1} \rangle \, \,,\,i > 1,$$

where $U_{enc} \left( \alpha \right)$ and $U\left( \theta \right)$ are the processing of single words in code gadgets. After $l_{\max }$ iterations of calculation, the measurement is performed to obtain the final output. $l_{\max }$ is the maximum length of the code gadget dataset.

The overall unitary transformation $U_{oval}$ is composed of $U_{sw}$. The relevant calculation formula is as follows:

$$\begin{aligned} U_{oval} = & \otimes_{i = 0}^{{l_{\max } }} \left( {U_{sw} \left( {\alpha_{i} ,\theta_{i} } \right)} \right) \\ =\, & U^{{\left( {l_{\max } } \right)}} \left( \theta \right)U_{{_{enc} }}^{{\left( {l_{\max } } \right)}} \left( \alpha \right) \cdots U^{\left( 2 \right)} \left( \theta \right)U_{{_{enc} }}^{\left( 2 \right)} \left( \alpha \right)U^{\left( 1 \right)} \left( \theta \right)U_{{_{enc} }}^{\left( 1 \right)} \left( \alpha \right). \\ \end{aligned}$$

The entire circuit return state is:

$$\begin{aligned} |\varphi \rangle =\, & U_{oval} H|\varphi_{0} \rangle \\ =\, & \otimes_{i = 0}^{{l_{\max } }} \left( {U_{sw} \left( {\alpha_{i} ,\theta_{i} } \right)} \right)|\varphi_{0} \rangle \\ =\, & U^{{\left( {l_{\max } } \right)}} \left( \theta \right)U_{enc}^{{(l_{\max } )}} \left( \alpha \right) \cdots U^{\left( 2 \right)} \left( \theta \right)U_{enc}^{(2)} \left( \alpha \right)U^{\left( 1 \right)} \left( \theta \right)U_{enc}^{(1)} \left( \alpha \right)|\varphi_{0} \rangle . \\ \end{aligned}$$

In our work, we apply a measurement in the Z-basis as the Hamiltonian. Finally, the expectation calculation formula is as follows:

$$\begin{aligned} E\left( Z \right) = & \langle \varphi |z \otimes z \otimes I \otimes \cdots \otimes I|\varphi \rangle \\ = & \langle \varphi_{0} H^{\dag } U_{oval}^{\dag } \rangle |z \otimes z \otimes I \otimes \cdots \otimes I|U_{oval} H\varphi_{0} \rangle . \\ \end{aligned}$$

Therefore, the model depth of the quantum neural network structure QDENN we propose for vulnerability detection depends on the maximum length of the dataset. The depth of the quantum circuit is *O(maxlen)*, *maxlen* is the maximum length of the code gadgets.

As for the classical neural network, the foremost is the word embedding using neural network or frequency statistics. This requires amounts of storage space to fully represent the semantic information, many works set the word embedding vector dimension to 128 and 256 usually. The input vector dimension of a classic recurrent neural network is $seqlen \times h_{vc}$, where $seqlen$ is the length of sequence and h_vc_ is the dimension of word embeddings. According to relevant research statistics^42^, the complexity per layer of recurrent is O(nd^2^) and convolutions is O(knd^2^), where k is the kernel size of convolutions.

In our work, can use $\log_{2} N$ qubits representing the dictionary consists of N words. The input vector dimension of QDENN is $\max len \times \log \left( N \right)$.

### Analysis of variable length data processing

Quantum neural networks can already be used for image classification, but they also have certain limitations. They can be roughly divided into two modes: 1. A quantum neural network is used to realize a convolutional layer or a fully connected layer, and it needs to work with a classical neural network. 2. A complete quantum neural network structure. If the first type is used, the advantages of quantum computing will not be realized. However, the second type often limits the input size of an image, which requires the image to be scaled first and then input to the quantum neural network. Regardless of the mode, image classification can scale an image to achieve the same length of data input to the quantum neural network.

However, Quantum neural networks can already be used for image classification, but they also have certain limitations. Especially in the field of vulnerability detection that this paper focuses on, the length of a vulnerability program has multiple styles, which also means that the length of the treatment is the key issue that needs to be solved next. To solve this problem, classical machine learning uses an LSTM neural network structure. At the same time, existing research has implemented a QLSTM^21,22^ model architecture based on VQC. However, they only combine the inapplicability of LSTM and VQC, which consumes considerable time. As been illustrated in the QLSTM, each QLSTM unit is implemented by 6 VQC circuits, and each VQC requires N measurements to obtain the QLSTM intermediate vector result, where N is the number of qubits required to encode the hidden layer vector into the quantum state. The principle of QLSTM is the same as the classic LSTM, and each word in the input sentence requires 1 calculation by the QLSTM unit. This means that if the input is a sentence of length L, which requires L calculations based on the QLSTM unit, a total of *L* $\times$ *6* $\times$ *N* measurement is required. The use of a hybrid classical and quantum structure undoubtedly offsets the advantages of quantum computing, but the time cost is extremely high. However, the QDENN model proposed in this paper only performs 2 measurements in the last measurement, which undoubtedly greatly reduces the number of measurements required.

Furthermore, in natural language processing, there have been related works based on quantum neural networks^26–29^. However, this part of the research is based on lexical analysis, which generates quantum circuits for each sentence based on tensor networks. However, this research can show considerable results in small-scale natural language processing tasks. However, in the field of large-scale vulnerability detection, vulnerability sentences are longer, and the lexical structure is more complex. Therefore, these methods are not suitable for vulnerability detection applications, and it is extremely critical to propose a set of neural networks that can be applied to vulnerability detection.

The QDENN structure proposed in this paper can solve the time consumption problem of a QLSTM model and can also solve the problem of batch processing with long sentences. To solve the problem of length inconsistency, this paper uses the maximum length of the dataset to fill the insufficient digits, and the filling character is the special <PAD> character. Although this method can effectively achieve the same length, the depth of the network structure of the QDENN is positively related to the maximum length of the dataset, which may also impact the accuracy rate.

### Experimental environment

Based on the method and our proposed QDENN quantum circuit, we employ SARD datasets^43^ to validate the correctness of QDENN software vulnerability detection. To increase code reliability and reproducibility, we conduct experiments based on the open-source program slicing in previous work^18^. Specifically, we divide the dataset according to the maximum length and conduct related experiments. We use $ml$ to represent the maximum length of the code gadgets and define the length interval of code gadgets as $\Delta r$. This helps us to explore the impact of adding padding data more fully on model performance. For example, the length of code gadgets in a certain set of data is limited between 40 and 70; then, we set $\Delta r = 30,ml = 70$. We randomly choose 5000 code gadgets generated from the programs as the dataset (50% of which are vulnerable and the remaining 50% are not), where $\Delta r = 10$ and $ml = 50,60,70,80,90$. Among them, the dataset is divided according to $ml$, and each interval contains 1000 code gadgets. For the sub dataset of $\Delta r = 30,40$, we obtain it by merging the datasets in the subintervals. In our work, we divide the training set and validation set using a ratio of 8:2.

Our experiment is conducted with the open-source Python framework, with mindspore as the employed quantum neural network and mindquantum for quantum circuit simulation. The biggest feature of mindspore is that it adopts the industry's latest source-to-source automatic differentiation, which can use the underlying technology of compilers and programming languages to further optimize and support better differential expression. We use the classical parameter optimizer as the SGD optimizer for the quantum neural network. For other hyperparameter settings, the momentum is set to 0.9, the weight decay is set to 0.0001, the batch size is set to 32, and the number of epochs is set to 10.

### Experimental analysis of model depth

In the QDENN model, the depth of the model is determined by the maximum length of the dataset. If the code gadgets of the dataset are too long, the depth of the QDENN model will be too deep. Therefore, we conduct related experiments to explore the impact of model depth changes brought about by different data intervals on the detection ability.

**Figure 4:**
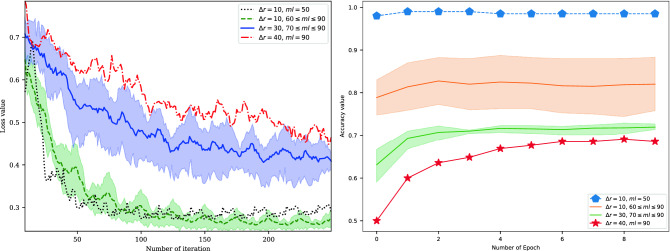
Training loss and validation accuracy values. Using the SARD datasets, we train QDENN models at different data intervals, with the batch size set to 32. The SGD optimizer is used as the parameter optimizer. To make the loss curve smoother, we process it based on the average value of a rolling window when drawing the curve, and the rolling window size is set to 3. Since the data intervals ($\Delta r$ is 30 and 40) are obtained by merging the subintervals, more iterations are required for training. In this study, we plot the training loss with 250 fixed iterations. Meanwhile, we plot the validation accuracy with the validation datasets under 10 epochs.

We confirm the training and validation statements for all data intervals with the experiment illustrated in Fig. 4. Using a cross-entropy loss function, training 10 epochs, we plot the training loss values and validate the accuracy values in Fig. 4. With the decrease in $\Delta r$, the loss value of the quantum neural network converges faster and can achieve lower loss. Under the condition of $\Delta r = 10$, the loss of the quantum neural network can be trained to the lowest loss value, which converges faster than other subintervals. With regard to the SARD datasets, the accuracy of the QDENN quickly converges to at least 98% with $\Delta r$ set to 10 and $ml$ set to 60. Conversely, with the increase in $ml$, the change in the depth of the model also affects the vulnerability detection accuracy of the quantum neural network. Specifically, it is negatively correlated.

To fully explore the boundary capabilities of our proposed model, we add padding data on the basis of a fixed-length interval and merge the related to obtain sub datasets with $\Delta r$ set to 30 and 40. It can be inferred that the padding data have a certain impact on the accuracy of the model, but the model is also robust to the padding dataset. With more padding data, the model can still learn the vulnerability characteristics and realize source code vulnerability detection.

Moreover, based on this experiment, we also have reason to believe that with the improvement of the quantum neural network model, under the influence of solving the depth of the model, the sensitive problem of the model’s padding data will also be solved.

### Comparison experiments with another quantum neural network

To demonstrate the novelty of our proposed QDENN, we conduct comparison experiments with another quantum neural network. In cutting-edge research^44–46^, the JQub team present a neural network and quantum circuit codesign framework named QuantumFlow. In the QuantumFlow framework, a new type of quantum awareness is designed to realize basic operations based on quantum circuits, including linear and nonlinear operations of vectors and batch normalization operations, named QF-Net. Moreover, QF-Circ is proposed to automatically generate and optimize a corresponding quantum circuit.

There have indeed been many quantum neural networks proposed to address specific problems. For example, QCNN and QuantumFlow, which have been demonstrated the effectiveness in image domain. Meanwhile, QLSTM proposed to combine the quantum neural networks with RNN structures. However, there is no research that combines quantum computing in the field of vulnerability detection. As described in the “The advantage of a quantum neural network” section, the QLSTM needs amounts of measurement, which greatly increases the time cost. Due to the excellent performance of QuantumFlow in quantum neural networks, we choose it as the baseline in this research.

**Figure 5:**
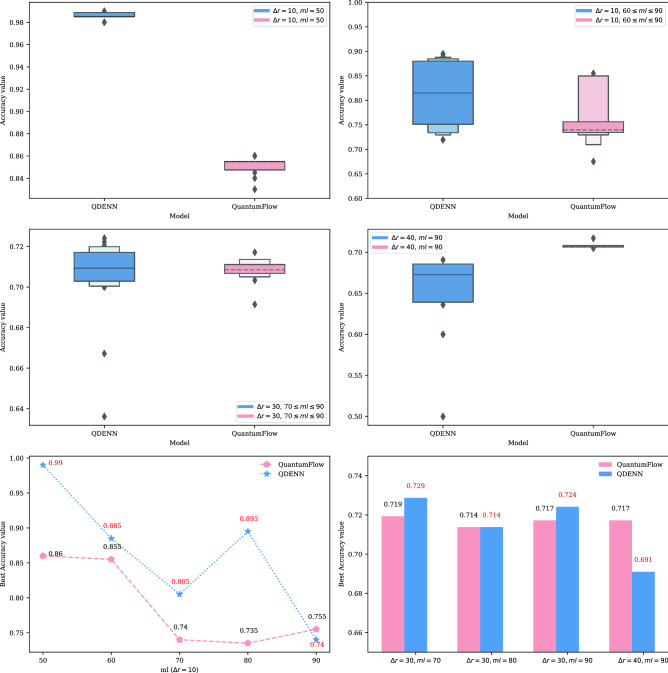
Validation accuracy value distribution during training. Here, the box plots reveal the distribution of accuracy values using different quantum neural networks during training. Meanwhile, the line chart and the histogram reveal the best accuracy comparison of the QDENN and QuantumFlow, which was chosen as the baseline in this study.

Different from our proposed QDENN structure, which performs quantum encoding on each vulnerability code word, QuantumFlow only supports quantum encoding of classical data with fixed qubits to form normalized quantum circuits. As mentioned above, vulnerability code gadgets, like other natural language data, are characterized by inconsistent sentence lengths. Moreover, the input to the vulnerability detection model is long code gadgets. Therefore, to apply QuantumFlow to the vulnerability detection field as a baseline for comparative experiments, we pretrain code gadgets of different lengths based on doc2vec^47^ to obtain fixed-length vectors. The method implements sentence embeddings based on classical neural networks and is trained for independent sentence embeddings. Therefore, intuitively speaking, the combined model based on doc2vec and QuantumFlow should have better vulnerability detection ability. However, this is false because the method of combining models can lead to an inappropriate combination of classical data and quantum encoding. The experimental results are illustrated in Fig. 5.

To ensure the credibility of the results, we adopt a dynamic learning rate adjustment strategy when training QuantumFlow. Specifically, we use the method of multi-step decay to adjust the learning rate. For hyperparameter settings, the milestones are set to^5,13,15^, gamma is set to 0.1 and initial learning rate is set to 0.01. In order to ensure the comparability of experiments, we also use SGD optimizer and a cross-entropy loss function to train 10 epochs.

We draw box plots based on the validated accuracy values of each epoch to show the fluctuations in the vulnerability detection capabilities of the QDENN and QuantumFlow. In the case of a lower model depth, the accuracy of the QDENN is higher than that of QuantumFlow, and it is more stable. However, when $\Delta r$ is 40, the training and verification accuracy and stability of the QDENN model are lower than those of QuantumFlow. At the same time, we draw a line chart and a histogram based on the best accuracy values of the models on each subinterval. Through the line chart, we can clearly see the influence trend of the ml value of the subinterval on the accuracy of the model. On the subinterval data, our proposed model has the best vulnerability detection effect, and the best accuracy rate is as high as 99%. On each subinterval data with $\Delta r$ is 10, the best average vulnerability detection accuracy of the model is 86.3%. Based on the QuantumFlow model, the best accuracy on the subinterval data of $\Delta r$ = 10 and $ml$ = 50 is 86%, and the overall best accuracy average with $\Delta r = 10$ is 78.9%.

Therefore, it can be inferred based on this experimental analysis that the vulnerability detection ability of our proposed QDENN is significantly better and more stable in the case of low depth. However, in the case of high depth, although the overall accuracy of the model is slightly higher than QuantumFlow, its stability is poor, and more epochs are required to train to the best performance.

## Discussion

We propose and demonstrate a new method based on a quantum neural network to detect vulnerability. In stark contrast to classical models, a quantum neural network structure can solve the memory bottleneck problem and has great potential. To the best of our knowledge, no such analysis has been performed for quantum neural networks.

To fill the gap in this field, we propose a quantum neural network structure named QDENN for vulnerability detection. This work is the first attempt to implement word embedding of vulnerability codes based on a quantum neural network, which proves the feasibility of a quantum neural network in the field of vulnerability detection. Compared to other quantum neural networks, our proposed QDENN can achieve higher vulnerability detection accuracy. On the sub dataset with a small-scale interval, the model accuracy rate reaches 99%. On each subinterval data, the best average vulnerability detection accuracy of the model reaches 86.3%. Meanwhile, our proposed QDENN can effectively solve the inconsistent input length problem of quantum neural networks and the problem of batch processing with long sentences.

This work attempts to address the gap in vulnerability detection but leaves room for further research. A feature map plays a large role in a quantum neural network. Different variational circuits can also influence the model’s landscape, which should be investigated. The accuracy of the quantum neural network could be further increased with a more sophisticated neural network architecture and training procedure. This work validates the use of a quantum neural network for vulnerability detection and opens a route for future applications.

Overall, we have shown that quantum neural networks can possess vulnerability detection—a promising revelation for quantum machine learning, which we hope leads to further studies on the power of quantum models.

## Data Availability

All the data that support the findings of this study are available from the corresponding authors (qf_zhouxin@126.com, jianmin_pang@126.com).
